# Peer Review of “Impact of COVID-19 Testing Strategies and Lockdowns on Disease Management Across Europe, South America, and the United States: Analysis Using Skew-Normal Distributions”

**DOI:** 10.2196/28743

**Published:** 2021-04-21

**Keywords:** COVID-19, testing strategy, skew-normal distributions, lockdown, forecast, modeling, outbreak, infectious disease, prediction

*This is a peer-review report submitted for the paper "COVID-19 Testing Strategies and Lockdowns: The European Closed Curves, Analyzed by Skew-Normal Distributions, Forecasts for the United Kingdom, Sweden, and the United States, and the Ongoing Outbreak in Brazil."*

## Round 1 Review

### General Comments

The title, abstract, and text of this manuscript [1] all aim to answer multiple questions pertaining to the dynamics and control of the COVID-19 pandemic in multiple regions/countries, using mathematical methods. However, neither of the questions nor the answers are clear, and the author has not completed any “translation” work, that is, translating mathematical calculation into descriptions, predictions, and control strategies of the pandemic.

My suggestion is that the author should focus on *one* specific issue about the COVID-19 pandemic; for example: when will adequate herd immunity be established in Sweden, the United Kingdom, and other European countries per the curves? Or how many people will die in the coming months in some European countries? Or which control strategy is the best or better for European countries? Then, the author should give a *clear* answer to the question through mathematical calculations.

## Round 2 Review

After reading the responses of the author to my comments, I read again with patience the manuscript, which actually has not been revised. I understand it is tough for a mathematical researcher to conduct this work as they need to acquire a lot of knowledge in virology, immunology, and infectious diseases. However, this manuscript really needs to be revised greatly for the following reasons:

1. In general, this manuscript is written in the style of lecture notes rather than a scientific report. For example, multiple tables were used without titles and legends, and none of the tables were in the standard format of a scientific report. Multiple tables could be deleted.
2. Per the author’s response, the main purpose of this paper was to prove that massive testing strategies are probably the best choice for managing the COVID-19 pandemic. Has this conclusion been given in the findings or interpretation discussed in the abstract (the answer is no)? Has this question been mentioned in the *Introduction* section (the answer is no)? What strategies are inferior to massive testing strategies; which are probably the best? Why and how should this conclusion be made through mathematical calculations?
3. The author should focus on the key question mentioned in the responses. The wordy explanation and calculation in the first three sections should be shortened. Otherwise, readers will not know what the author wants to convey using mathematical language.

The author could consider the following structure: the COVID-19 situation; the question this paper will answer; the effects of the control measures (eg, distancing) that will be involved in the mathematical model; the roles of the parameters (eg, TCCpM) that will be involved in the mathematical model; the principle of the mathematical model; the mathematical model; the answer to the target question through calculation using the model and the epidemiological data.
